# Ofatumumab maintenance prolongs progression-free survival in relapsed chronic lymphocytic leukemia: final analysis of the PROLONG study

**DOI:** 10.1038/s41408-019-0260-2

**Published:** 2019-12-04

**Authors:** Marinus van Oers, Lukas Smolej, Mario Petrini, Fritz Offner, Sebastian Grosicki, Mark-David Levin, Jaclyn Davis, Hiya Banerjee, Tommaso Stefanelli, Petra Hoever, Christian Geisler

**Affiliations:** 1 Academisch Medisch Centrum and HOVON, Amsterdam, The Netherlands; 2 University Hospital and Faculty of Medicine, Hradec Kralove, Czech Republic; 3 Azienda Ospedaliero Universitaria Pisana, Pisa, Italy; 4 Universitair Ziekenhuis Gent, Gent, Belgium; 5 Department of Hematology and Cancer Prevention, Silesian Medical University, Katowice, Poland; 6 Albert Schweitzer Ziekenhuis Dordrecht and HOVON, Dordrecht, The Netherlands; 7 Novartis Oncology, East Hanover, NJ USA; 8 Novartis Pharma AG, Basel, Switzerland; 9 Rigshospitalet-Koebenhavn, Copenhagen, Denmark

**Keywords:** Lymphoma, Cancer therapy

## Abstract

We report the final analysis of the PROLONG study on ofatumumab maintenance in relapsed chronic lymphocytic leukemia (CLL). In all, 480 patients with CLL in complete or partial remission after second- or third-line treatment were randomized 1:1 to ofatumumab (300 mg first week, followed by 1000 mg every 8 weeks for up to 2 years) or observation. Median follow-up duration was 40.9 months. Median progression-free survival was 34.2 and 16.9 months for ofatumumab and observation arms, respectively, (hazard ratio, 0.55 [95% confidence interval, 0.43–0.70]; *P* < 0.0001). Median time to next treatment for ofatumumab and observation arms, respectively, was 37.4 and 27.6 months (0.72 [0.57–0.91]; *P* = 0.0044). Overall survival was similar in both arms; median was not reached (0.99 [0.72–1.37]). Grade ≥ 3 adverse events occurred in 62% and 51% of patients in ofatumumab and observation arms, respectively, the most common being neutropenia (23% and 10%), pneumonia (13% and 12%) and febrile neutropenia (6% and 4%). Up to 60 days after the last treatment, four deaths were reported in the ofatumumab arm versus six in the observation arm, none considered related to ofatumumab. Ofatumumab maintenance significantly prolonged progression-free survival in patients with relapsed CLL and was well tolerated.

## Introduction

As of 2018, chronic lymphocytic leukemia (CLL) remains an incurable disease. Therefore, prolonged progression-free survival (PFS) and overall survival (OS) with good quality of life remain the most important treatment goals. Improvement in PFS can result from more effective induction treatment, and further prolongation may be obtained by maintenance therapy. In 2015, we published results of a planned interim analysis of PROLONG, an open-label, randomized, phase III study that evaluated ofatumumab, a human type I CD20 monoclonal antibody (mAb), as maintenance treatment in patients with CLL who are in at least partial remission (PR) after induction treatment for relapse. Major conclusions were that ofatumumab maintenance compared with observation resulted in significant improvement in PFS and was well tolerated^1^. Here we report the final analysis results for key primary and secondary endpoints of the study. Final analysis was triggered by protocol-defined 280 investigator-assessed PFS events.

## Materials and methods

### Study design and patients

PROLONG (*ClinicalTrials.gov identifier: NCT01039376*) was an open-label, randomized, phase III study conducted at 130 centers in 24 countries in patients with CLL in complete remission (CR) or PR (according to the International Workshop on CLL [IWCLL] updated National Cancer Institute-Working Group [NCI-WG] guidelines^2^) after second- or third-line treatment. The inclusion and exclusion criteria have been previously described^1^. The study was conducted according to the Declaration of Helsinki and Good Clinical Practice guidelines and approved by the institutional review boards of all participating institutions. Written informed consent has been obtained.

### Randomization and treatment

Randomization (1:1) to ofatumumab or observation was stratified by response at entry (CR or PR), number of previous induction treatments (2 or 3) and type of the most recent treatment (chemoimmunotherapy, alkylating monotherapy or other treatment). Crossover was not allowed between study arms.

Within 1 week of treatment assignment, patients in the ofatumumab arm started with intravenous 300 mg treatment, followed 1 week later by 1000 mg every 8 weeks for up to 2 years. Dose reductions were not allowed; however, interruption or delay of administration because of adverse events (AEs) was permitted. Patients were treated until disease progression, withdrawal from study treatment due to unacceptable AEs, consent withdrawal or other reasons. Patients who prematurely discontinued study treatment were included in the analysis regardless of treatment duration.

### Assessment of efficacy and safety

Study parameters assessed at study entry and during follow-up have been previously described^1^. AEs were measured using the National Cancer Institute Common Terminology Criteria for Adverse Events version 4.0.

The primary endpoint was investigator-assessed PFS, defined as the time from randomization to the earliest date of disease progression or death due to any cause. For patients who did not progress or die, PFS was censored at the time of last adequate assessment. In addition, PFS was censored for patients who received new anticancer treatment before disease progression and for those with 2 or more missing assessments. Secondary endpoints included OS, time to next treatment (TTNT), PFS after next-line therapy (defined as the time from randomization until progression or death following next-line therapy) and safety. Responses were defined and assessed according to IWCLL updated NCI-WG guidelines^2^.

### Statistical analysis

Final analysis was conducted when a minimum of 280 events of disease progression or death occurred, which was needed to detect the targeted 40% improvement in PFS (hazard ratio [HR], 0.71) difference with 80% power and 5% two-sided α level. Efficacy analyses were conducted on the intent-to-treat (ITT) population regardless of the actual treatment received. Safety analysis was based on the actual treatment received. PFS, OS, TTNT and PFS after next-line therapy were analyzed using a stratified log-rank test adjusted for stratification factors. Kaplan–Meier curves were generated to determine differences between survival distributions of the treatment arms. All *P* values are two-sided. Statistical analyses were performed using SAS software (version 9.3; SAS Institute, Cary, NC, USA).

## Results

### Patients and treatment

**Fig. 1:**
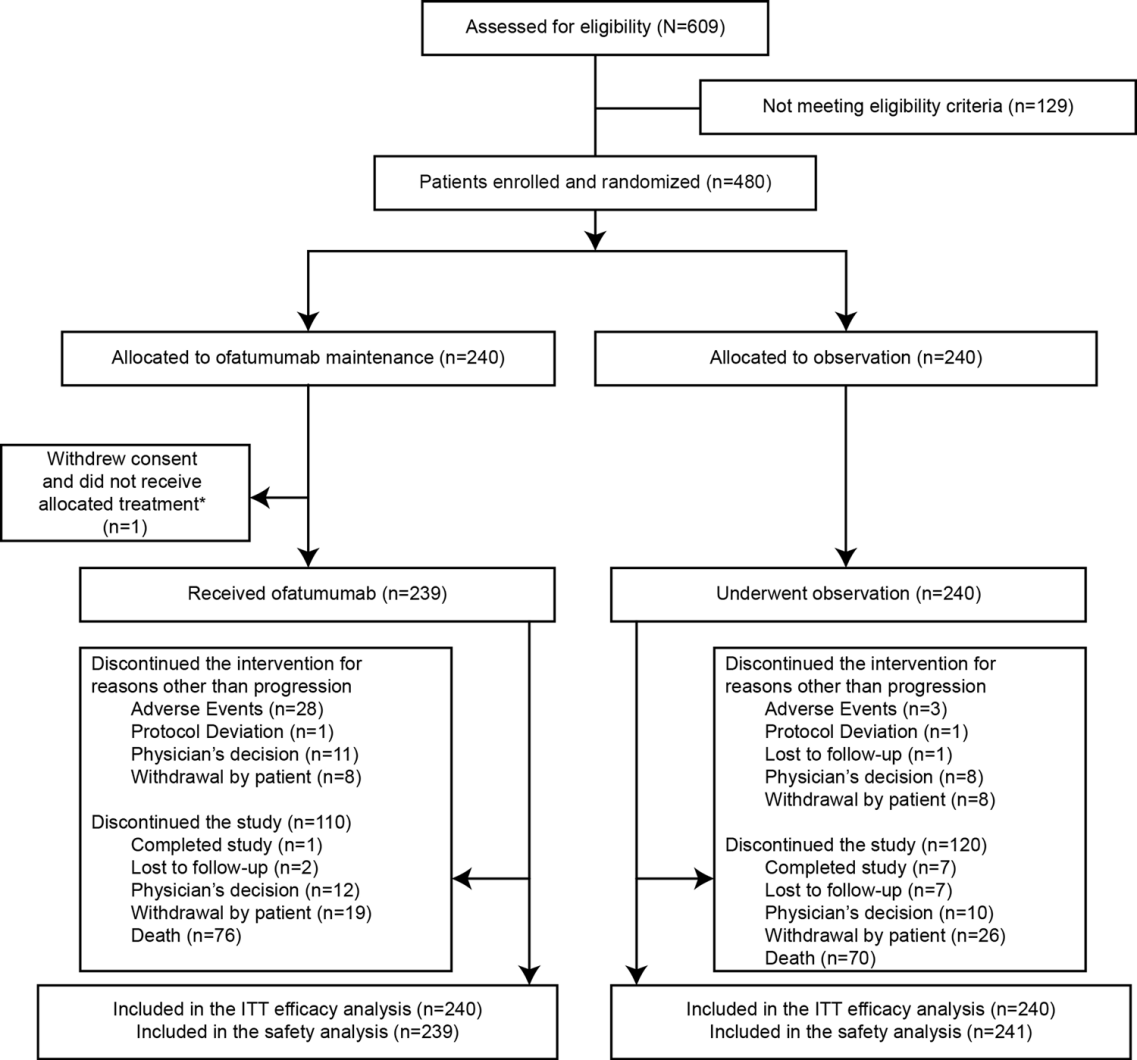
PROLONG study CONSORT diagram. ITT intent-to-treat.

Between 31 May 2010 and 01 October 2014, 609 patients were screened. A total of 480 patients, including six additional patients enrolled since interim analysis, were randomized to ofatumumab maintenance (*n* = 240) or observation (*n* = 240) (Fig. 1). All 480 patients were included in ITT analyses. One patient from the ofatumumab arm did not receive the allocated intervention (withdrew consent) and was therefore included in the observation arm for safety analysis. At the time of final analysis (cutoff date, 20 February 2017), 146 (30.5%) patients had died, and disease progression had occurred in 267 (56%) patients (Fig. 1).

Baseline demographics and clinical characteristics were well balanced between the study arms (Table 1). In total, 385 (80%) patients were in PR, 94 (20%) were in CR and 1 (<1%) had missing data. Overall, 337 (70%) patients had received 2 prior treatments. The most recent treatment was chemoimmunotherapy in 386 (80%) patients, primarily fludarabine, cyclophosphamide and rituximab (53%) and bendamustine and rituximab (24%), while only 23 (5%) patients had received alkylating monotherapy (Table 1); of note, 403 (84%) patients had previously received a rituximab-containing treatment regimen.

**Table 1:** Demographics and baseline disease characteristics^a^.

	Ofatumumab (*n* = 240)	Observation (*n* = 240)
Age, years^b^		
Median (min–max)	64.0 (33–86)	64.5 (39–87)
<70, *n* (%)	167 (70)	166 (69)
≥70, *n* (%)	73 (30)	74 (31)
≥75, *n* (%)	42 (18)	35 (15)
Sex, *n* (%)		
Female	79 (33)	80 (33)
Male	161 (67)	160 (67)
Time since diagnosis, median (range) in year	6.0 (1–22)	5.0 (1–22)
Response to last CLL treatment, *n* (%)		
CR	47 (20)	47 (20)
PR	193 (80)	192 (80)
Missing	0	1 (<1)
Baseline MRD, *n* (%)		
Negative	31 (13)	42 (18)
Positive	139 (58)	107 (45)
Missing	70 (29)	91 (38)
No. of prior treatments, *n* (%)		
2	169 (70)	168 (70)
3	67 (28)	63 (26)
Other	4 (2)	9 (4)
Type of last prior treatment, *n* (%)		
Chemoimmunotherapy	193 (80)	193 (80)
BR	46 (24)	48 (25)
FCR	100 (52)	105 (54)
FR	4 (2)	5 (3)
Other	30 (16)	24 (12)
RCVP	13 (7)	11 (6)
Alkylating monotherapy	14 (6)	9 (4)
Other	33 (14)	38 (16)
Baseline cytogenetics, *n* (%)^c^		
11q deletion	15 (6)	13 (5)
17p deletion	7 (3)	4 (2)
6q deletion or 12q trisomy or 13q deletion	47 (20)	16 (7)
No aberration	151 (63)	174 (73)
Missing	20 (8)	33 (14)
IGVH mutational status, *n* (%)		
Mutated	54 (23)	74 (31)
Unmutated	139 (58)	116 (48)
Not available	3 (1)	1 (<1)
Missing	44 (18)	49 (20)

*BR* bendamustine and rituximab, *CLL* chronic lymphocytic leukemia, *CR* complete remission, *FCR* fludarabine, cyclophosphamide, and rituximab, *FR* fludarabine and rituximab, *IGVH* immunoglobulin variable heavy-chain gene, *ITT* intent-to-treat, *MRD* minimal residual disease, *PR* partial remission, *RCVP* rituximab, cyclophosphamide, vincristine, and prednisone

^a^ITT population

^b^Age was calculated from birth date to screening date in years

^c^12% cutoff

Overall, 186 (78%) patients received 100% and 42 (18%) received 80% to <100% of the assigned ofatumumab dose. Only 11 (5%) patients received <80% of the expected total ofatumumab dose. Primary reasons for ofatumumab discontinuation included AEs (12%), the most frequent being neutropenia (2%), refusal/withdrawal by patient (3%), physician decision (5%) and protocol deviation (<1%; Fig. 1).

### Efficacy

**Fig. 2:**
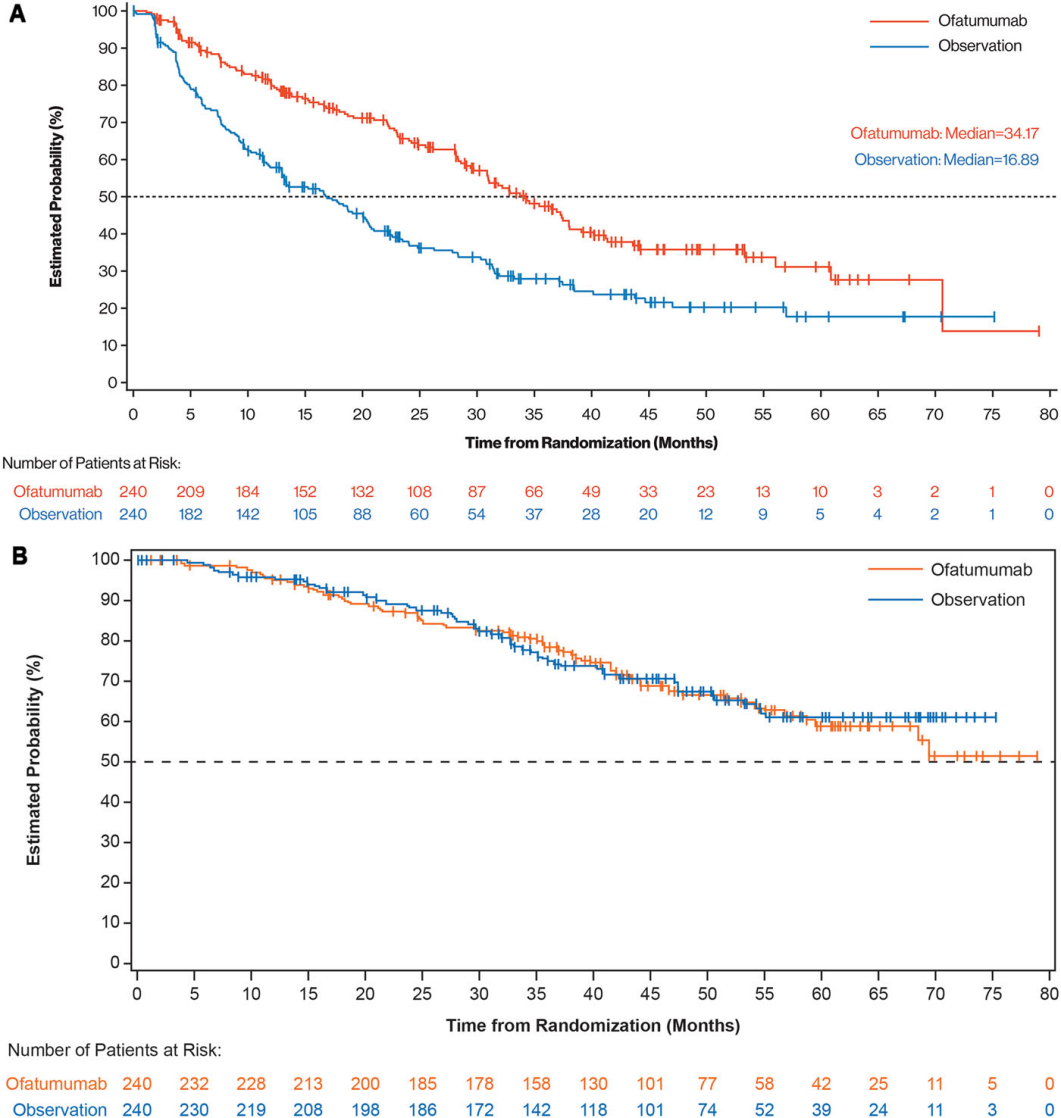
ITT analysis of **a** PFS, as assessed by investigator, and **b** OS. ITT: intent-to-treat; OS: overall survival; PFS: progression-free survival.

**Fig. 3:**
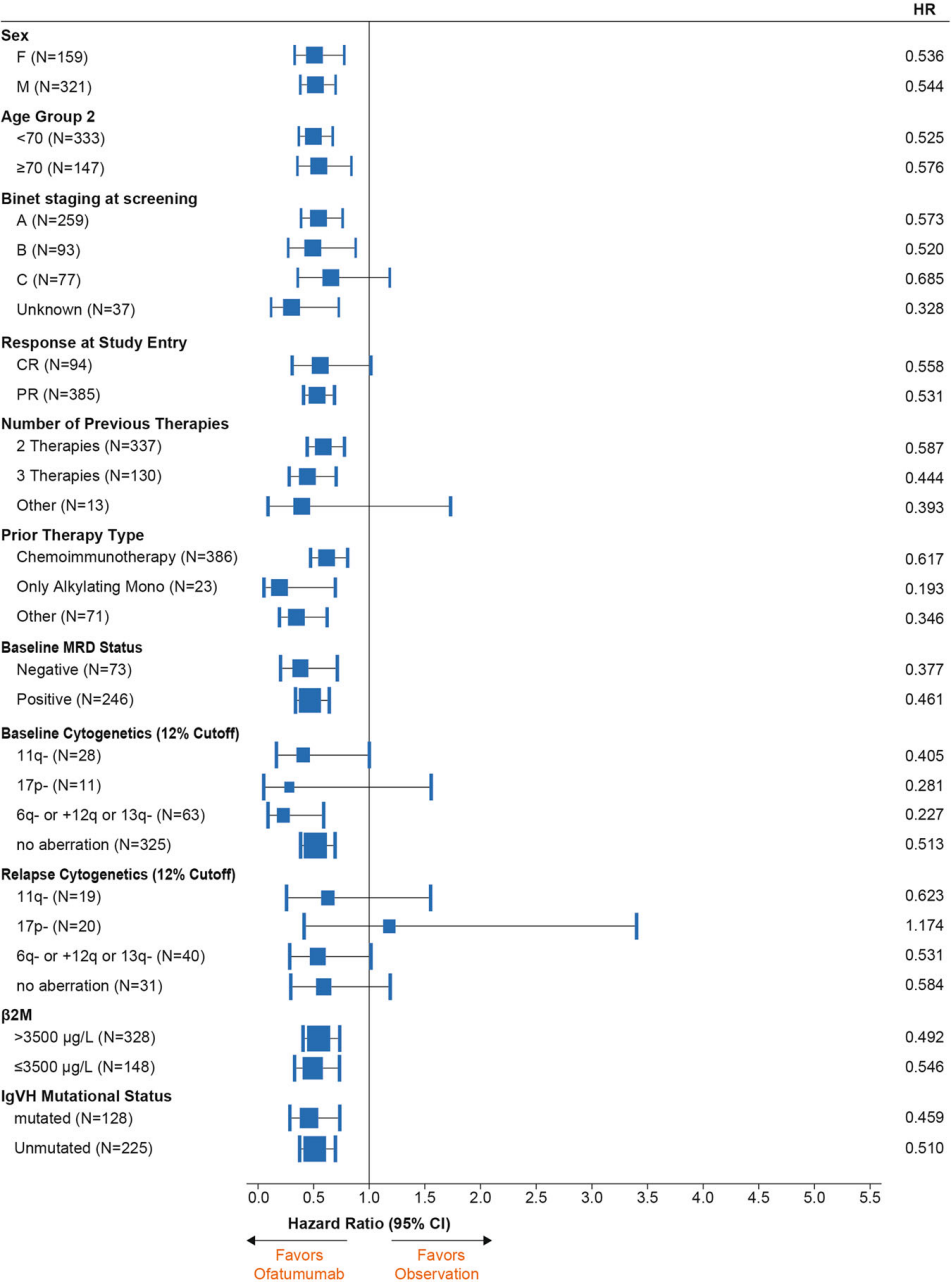
Forest plot of subgroup analysis of PFS. β2M β_2_-microglobulin; CI confidence interval; CR complete remission; HR hazard ratio; *IGVH* immunoglobulin variable heavy-chain gene; MRD minimal residual disease; PFS progression-free survival; PR partial remission.

At the time of final analysis, the median follow-up duration was 40.9 months. Compared with observation, ofatumumab maintenance resulted in significant and clinically relevant improvement in the primary endpoint. Investigator-assessed PFS was 34.2 months (95% confidence interval [CI], 29.7–38.0) for the ofatumumab arm versus 16.9 months (95% CI, 13.0–20.4) for the observation arm (HR, 0.55 [95% CI, 0.43–0.70]; *P* < 0.0001; Fig. 2a). Assessment of PFS by the independent review committee yielded similar results: 33.6 months (95% CI, 28.0–37.2) for the ofatumumab arm versus 15.0 months (95% CI, 11.4–19.0) for the observation arm (HR, 0.57 [95% CI, 0.46–0.72]; *P* < 0.0001). When events detected by computed tomography scan were also included, the investigator-assessed PFS was shorter (ofatumumab maintenance 27.5 months [95% CI, 23.0 to 30.2] versus observation 13.1 months [95% CI, 11.6–16.9]; HR, 0.64 [95% CI, 0.52–0.78]; *P* < 0.0001). The improved PFS did not translate into a difference in OS, and median OS was not reached in both arms (HR, 0.99 [95% CI, 0.72–1.37]; Fig. 2b). As shown in the forest plots (Fig. 3), the PFS benefit was evident in most subgroups, including baseline demographic characteristics (age and gender), response at study entry, type of prior therapy, baseline minimal residual disease (MRD) (Supplementary Fig. 1) and immunoglobulin variable heavy-chain gene (*IGVH*) mutation status. A clear exception was patients with 17p deletion at relapse on study who did not benefit from ofatumumab maintenance treatment. Although patients with 17p deletion at baseline (i.e., at study entry) seem to benefit from ofatumumab maintenance, this group is very small, precluding robust conclusions. Unfortunately, results of cytogenetic testing performed at the time of original CLL diagnosis were not available.

**Fig. 4:**
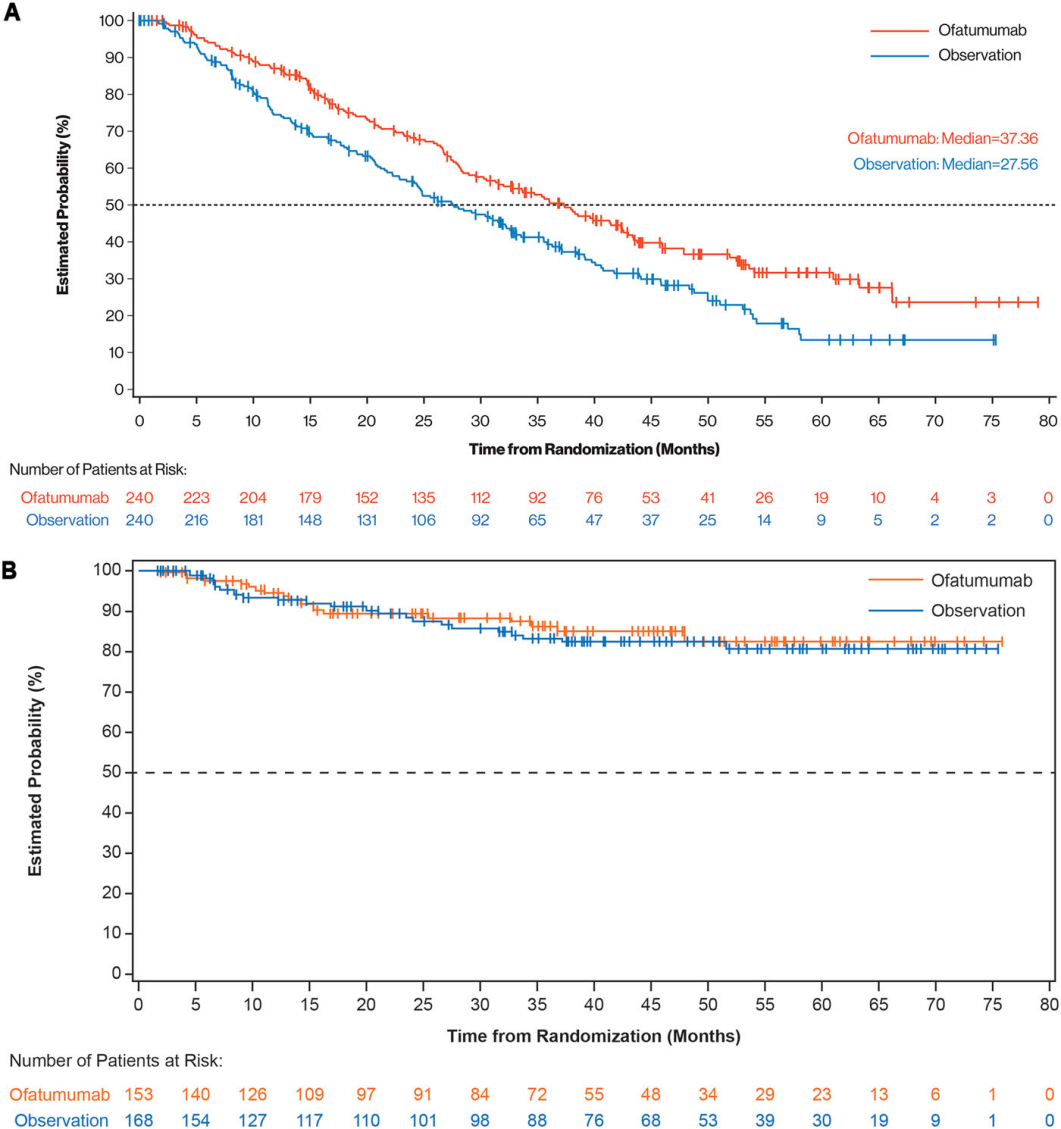
Time to next-line treatment and PFS after next-line treatment. **a** TTNT and **b** PFS after next-line treatment, defined as the time from randomization to the second disease progression or death from any cause, in patients receiving next-line treatment for relapse in the study. CLL chronic lymphocytic leukemia; PFS progression-free survival; TTNT time to next treatment.

Subsequent treatment was administered to 133 (55%) patients in the ofatumumab arm and 155 (65%) patients in the observation arm. Ofatumumab maintenance improved TTNT compared with observation (37.4 months [95% CI, 30.6–42.6] versus 27.6 months [95% CI, 23.5–32.6], respectively; HR, 0.72 [95% CI, 0.57–0.91]; *P* *=* 0.004; Fig. 4a). PFS after next-line therapy was defined as the time from randomization to the second objective disease progression or death from any cause, whichever occurred first. Patients who did not progress or die after next-line therapy were censored at their last date of contact. PFS after next-line treatment was not different between the two arms (median not reached; HR, 0.88 [95% CI, 0.49–1.61]; Fig. 4b). Steroids and some antineoplastic agents (i.e., rituximab, cyclophosphamide, chlorambucil) were used slightly more frequently for next treatment in the observation arm. Interestingly, follow-up therapy for the ofatumumab arm versus the observation arm comprised ofatumumab for 1 (<1%) versus 15 (9%) re-treated patients, idelalisib for 5 (3%) versus 11 (7%) patients, ibrutinib for 54 (35%) versus 48 (29%) patients, acalabrutinib for 2 (1.3%) versus 0 (0%) patients and venetoclax for 4 (3%) versus 3 (2%) patients, respectively. Thus, about 40% of patients received novel agents as next-line treatment.

### Toxicity

Both the total number of AEs and number of grade ≥ 3 AEs were higher in the ofatumumab arm than in the observation arm (Table 2). An increased incidence of grade ≥ 3 neutropenia was observed in the ofatumumab arm compared with the observation arm (23% [56/239] versus 10% [24/241]). Prolonged and severe neutropenia, defined as grade 3 or 4 neutropenia occurring during the treatment period and not resolved at least 42 days after the last dosing date, occurred in 11/239 (5%) patients in the ofatumumab maintenance arm and 4/241 (2%) patients in the observation arm. The increased incidence of grade ≥ 3 neutropenia likely contributed to the observed increase in grade ≥ 3 infections in patients in the ofatumumab (31% [73/239]) versus observation (25% [60/241]) arms. The use of growth factor support was reported 58 times among the 239 patients in the ofatumumab maintenance safety population versus 24 times in the 241 patients in the observation arm. At study entry, in both study arms, median immunoglobulin (Ig) A and IgM levels were decreased, whereas IgG levels were just above the lower limit (Supplementary Fig. 2). During the treatment phase, IgM, IgG, and IgA levels did not change significantly in the ofatumumab arm. In contrast, in the observation arm, serum IgM levels gradually increased to just above the lower limit of normal, whereas IgG and IgA levels did not change. During follow-up, IgM levels showed a slight increase in the ofatumumab arm. Unfortunately, beyond 30 months of follow-up, these small values preclude robust conclusions. Peripheral blood B cells began recovering 3 months after the end of ofatumumab maintenance (data not shown).

**Table 2:** Any AEs in ≥2% (grade ≥ 3) of patients by haematologic toxicity and infections.

	All Grade		Grade ≥ 3	
Preferred term	Ofatumumab (*n* = 239)	Observation (*n* = 241)	Ofatumumab (*n* = 239)	Observation (*n* = 241)
Any event, *n* (%)	209 (87)	168 (70)	105 (44)	74 (31)
Haematologic toxicity, *n* (%)				
Neutropenia	64 (27)	27 (11)	56 (23)	24 (10)
Febrile neutropenia	17 (7)	11 (5)	14 (6%)	9 (4%)
Thrombocytopenia	14 (6)	15 (6)	5 (2)	8 (3)
Anaemia	9 (4)	15 (6)	5 (2)	7 (3)
Neutrophil count decreased	8 (3)	3 (1)	5 (2)	2 (<1)
Infections, *n* (%)				
Pneumonia	42 (18)	41 (17)	32 (13)	28 (12)
Pyrexia	51 (21)	31 (13)	12 (5)	6 (2)
Sepsis	7 (3)	5 (2)	7 (3)	5 (2)
Septic shock	5 (2)	1 (<1)	5 (2)	1 (<1)
Lung infection	4 (2)	4 (2)	4 (2)	3 (1)
Upper respiratory tract infection	54 (23)	28 (12)	4 (2)	1 (<1)
Herpes zoster	17 (7)	12 (5)	3 (1)	4 (2)
Urinary tract infection	13 (5)	12 (5)	2 (<1)	5 (2)
Cellulitis	5 (2)	5 (2)	2 (<1)	4 (2)
Respiratory tract infection	18 (8)	18 (7)	2 (<1)	4 (2)
Infusion-related reaction, *n* (%)	42 (18)	0	3 (1)	0

AEs as reported by the investigator

Infusion-related reactions were defined as events occurring during infusion or within 24 h after completion of infusion and included chills, dyspnea, flushing, hypotension, nausea, pain, pruritus, pyrexia, rash, and urticaria

*AE* adverse event

Only 4% (9/239) of patients experienced grade ≥ 3 infusion-related AEs, which were defined as events occurring during infusion or within 24 h after completion of infusion, which the investigator attributed to the treatment medication. These events included, but were not limited to, chills, dyspnea, flushing, hypotension, nausea, pain, pruritus, pyrexia, rash and urticaria. AEs that led to treatment discontinuation occurred in 12% (28/239) of patients in the ofatumumab arm.

During the period from the first dose to 60 days after the last dose, four deaths were reported in the ofatumumab arm (one event each of pneumonia, cerebral haemorrhage, sepsis and small bowel obstruction) and six in the observation arm (two subdural haematoma, one fever and gastric pain, one intestinal infarction, one cardiac arrest, and one disease progression). None of these deaths were attributed to the study drug. At the time of final analysis, a total of 146 (30%) patients had died: 76 (32%) in the ofatumumab arm and 70 (29%) in the observation arm. Disease under study was the most frequent cause of death, with 46 (19%) deaths in the ofatumumab arm and 35 (15%) in the observation arm. Fatal serious AEs occurred in 26 (11%) patients in the ofatumumab arm and 33 (14%) patients in the observation arm. Of note, extremely few occurrences of Richter transformation (none in the ofatumumab arm versus 2 in the observation arm) were observed.

## Discussion

Final analysis of the PROLONG study confirmed the interim analysis results^1^ and showed that ofatumumab maintenance improved both PFS and TTNT in patients with relapsed CLL and was well tolerated. The PFS benefit was independent of baseline demographic characteristics, remission status at study entry, prior treatments and *IGVH* mutation status. Ofatumumab maintenance was accompanied by an increased risk of grade 3/4 neutropenia and grade 3/4 infections. Importantly, ofatumumab maintenance did not increase the risk of Richter transformation nor did it impair the response to next-line treatment.

Although the values are relatively small and our study was not powered to compare these subgroups, the benefit of ofatumumab seems more pronounced in the MRD-negative subgroup (Supplementary Fig. 1). This is an important finding because it shows that patients with good response to induction therapy may benefit from additional maintenance therapy. Greil et al. did not find any significant improvement in PFS with rituximab maintenance in patients with CLL who were MRD negative after first- or second-line treatment^3^. Results of long-term follow-up in their lesser pre-treated patient group would certainly be of interest.

Analyses of the cytogenetic subgroups (Fig. 3) should be interpreted with caution because of the very low number of patients with positive testing; this is most likely because patients were in PR or CR at study entry, resulting in levels of cytogenetic abnormalities below the detection threshold, notably in MRD-negative patients. This implies that the group without cytogenetic abnormalities harbors patients with chromosomal aberrations. Unfortunately, data on cytogenetics before study entry were not available. In addition, the low number of patients with 17p deletion might be partially attributable to the fact that these patients probably would not have been considered for participation in our study because they were candidates for other treatment modalities, such as allogeneic stem cell transplantation or treatment with kinase inhibitors. The conclusion that the PFS benefit with ofatumumab maintenance is independent of *IGVH* mutation status was based on an analysis of a robust number of patients, despite 26% of patients missing data in both study arms.

In our study, the interval between progression and next treatment appeared to be longer in the observation arm than in the maintenance arm (Figs. 2a and 4a). More rapid disease progression due to a possible difference in the biology of relapse was not supported by the observed similar PFS after next-line treatment in both study arms (Fig. 4b). Alternatively, lymphocytosis (not necessitating rapid treatment) might be a more frequent mode of progression in the observation arm than in the maintenance arm as long as there is circulating antibody. However, this is not supported by peripheral lymphocyte counts at relapse, which, although slightly lower in the maintenance arm, were not different between the two study arms (Supplementary Table 1). Because CLL progression as such is not an indication to start next treatment, TTNT is susceptible to subjectivity and, therefore, a less robust endpoint compared with PFS. Whether the threshold for initiation of treatment for progression during or after maintenance versus observation was different is speculative. PFS after next-line treatment (consisting of kinase inhibitors in ~40% of patients) in this cohort with 2 or 3 prior treatments is quite remarkable and reflects the progress that has been made in treatment of patients with relapsed CLL.

With longer follow-up, toxicity data were very similar to those found in the interim analysis^1^. Ofatumumab maintenance was associated with an increase in grade ≥ 3 neutropenia and infections, which was comparable to findings from studies on maintenance therapy with the chimeric anti-CD20 mAb rituximab in both follicular lymphoma^4^ and CLL^3^. No unexpected toxicity was observed. We have previously shown that no clinically relevant differences in health-related quality of life between the two study arms at any time point during treatment were observed^1^.

At the time of interim analysis in 2014, results of only one randomized phase III trial of maintenance treatment in CLL had been published^1^. In a study of 201 newly diagnosed or previously treated patients with CLL, rituximab maintenance for 2 years was found to improve PFS and OS but only in high-risk (11q and 17p) patients^5^. Since then, four other randomized phase III studies have been reported. First, final analysis of a multicenter trial on rituximab maintenance for 2 years in 263 patients with CLL (77% after first-line and 23% after second-line treatments) showed a median PFS of 47 months in the rituximab arm versus 35.5 months in the observation arm (HR, 0.50 [95% CI, 0.33–0.75]; *P* = 0.0007). Median OS was not reached in either arm. Grade 3 to 4 neutropenia occurred in 21% of patients in the rituximab arm versus 11% in the observation arm, with grade ≥ 3 infections in 20% versus 10% of patients, respectively^3^. Second, in a randomized, double-blind, placebo-controlled, phase III trial conducted in 314 previously treated patients with CLL, lenalidomide maintenance given until disease progression improved median PFS from 9.2 months in the observation arm to 33.9 months in the lenalidomide arm (HR, 0.40 [95% CI, 0.29 to 0.55]; *P* < 0.0001) but did not improve OS. The most common grade 3 or 4 AEs were neutropenia (lenalidomide arm [60%] versus placebo arm [23%]) and thrombocytopenia (17% versus 6%)^6^. Third, a randomized, double-blind, phase III study was performed in 89 high-risk patients with CLL, defined as those having post induction intermediate or high MRD levels combined with an unmutated *IGVH* gene status or *TP53* alterations. Lenalidomide maintenance after first-line therapy administered until disease progression improved the median PFS (13.3 months in the placebo arm versus not reached in the lenalidomide arm; HR, 0.168 [95% CI, 0.074–0.379]; *P* < 0.0001) but not OS. Grade ≥ 3 neutropenia occurred in 60% of patients in the lenalidomide arm versus 23% in the control arm^7^. In a French study, 409 treatment-naïve and fit patients with CLL aged ≥ 65 years in CR or PR after induction with four cycles of fludarabine, cyclophosphamide and rituximab were randomized to rituximab maintenance or observation. Median PFS in the rituximab arm (59.3 months) was greater compared with the observation arm (49.0 months) (HR, 0.55; *P* = 0.0002). Neutropenia and grade 3 to 4 infections were more common with rituximab maintenance versus observation (53% versus 36 and 19% versus 10%, respectively)^8^.

Taken together, these six randomized phase III studies, four of which included an anti-CD20 mAb, clearly demonstrate the clinical benefit of maintenance treatment in CLL. In all these studies maintenance treatment was applied after induction treatment with chemoimmunotherapy. One might argue that our data are less relevant in the present era of promising data on the use of novel agents in CLL, notably Bruton’s tyrosine kinase inhibitors^9,10^, phosphatidylinositol-3-kinase inhibitors^11^ and BCL-2 antagonists^12,13^. Indeed, depending on the outcome of ongoing trials in previously untreated patients comparing “chemofree” induction regimens consisting of (a combination of) novel agents with induction treatment with chemoimmunotherapy, it is expected that upfront use of chemoimmunotherapy in high-risk CLL patients will decrease. However, at present the possible role of chemoimmunotherapy in patients relapsing after prior use of (multiple) novel agents is unclear. Thus for relapsed patients (the population investigated in our trial) our findings might still be relevant. Importantly, thus far the novel agents are all recommended to be continued until relapse, leading to a very prolonged maintenance therapy, despite the limited availability of robust data on long-term safety, including risk of infections, emergence of mutated resistant clones^14,15^ and Richter transformation^16^. Results of ongoing studies on efficacy of kinase inhibitors and BCL-2 antagonists when used for a more restricted period would be relevant for assessment of optimal use of these drugs. In this setting, head-to-head comparisons of efficacy and safety of (prolonged) maintenance therapy with novel drugs versus anti-CD20 mAbs such as ofatumumab are relevant to determine optimal maintenance strategies for patients with CLL. In such studies, quality of life and cost-effectiveness should be important secondary endpoints.

## Supplementary information

Supplementary data
