# Supplementary material for: Ofatumumab maintenance prolongs progression-free survival in relapsed chronic lymphocytic leukemia: final analysis of the PROLONG study
Source: Blood Cancer J. 2019 Dec 4;9(12):98. doi: 10.1038/s41408-019-0260-2 (PMC6893027; doi:10.1038/s41408-019-0260-2)
Supplement: Supplementary file 1 — Supplementary data [file 41408_2019_260_MOESM1_ESM.pdf]

Supplemental Figure 1. PFS by baseline MRD status

(A) MRD-negative and (B) MRD-positive

Supplemental Figure 2. Immunoglobulin levels: (A) IgM, (B) IgG, and (C) IgA

Horizontal green lines indicate lower and higher limits of normal

Figure S1

A)

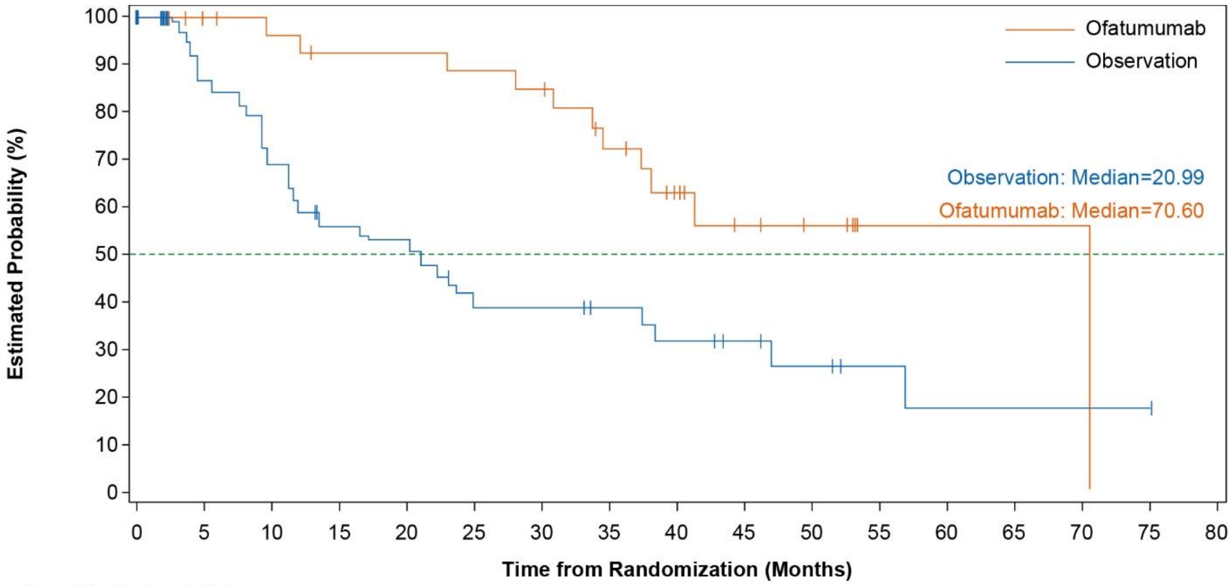

Number of Subjects at Risk:

	0	5	10	15	20	25	30	35	40	45	50	55	60	65	70	75	80
Ofatumumab	31	28	26	24	24	23	22	17	11	7	4	1	1	1	1	0	0
Observation	42	34	27	20	19	13	13	11	9	7	5	3	2	2	2	1	0

B)

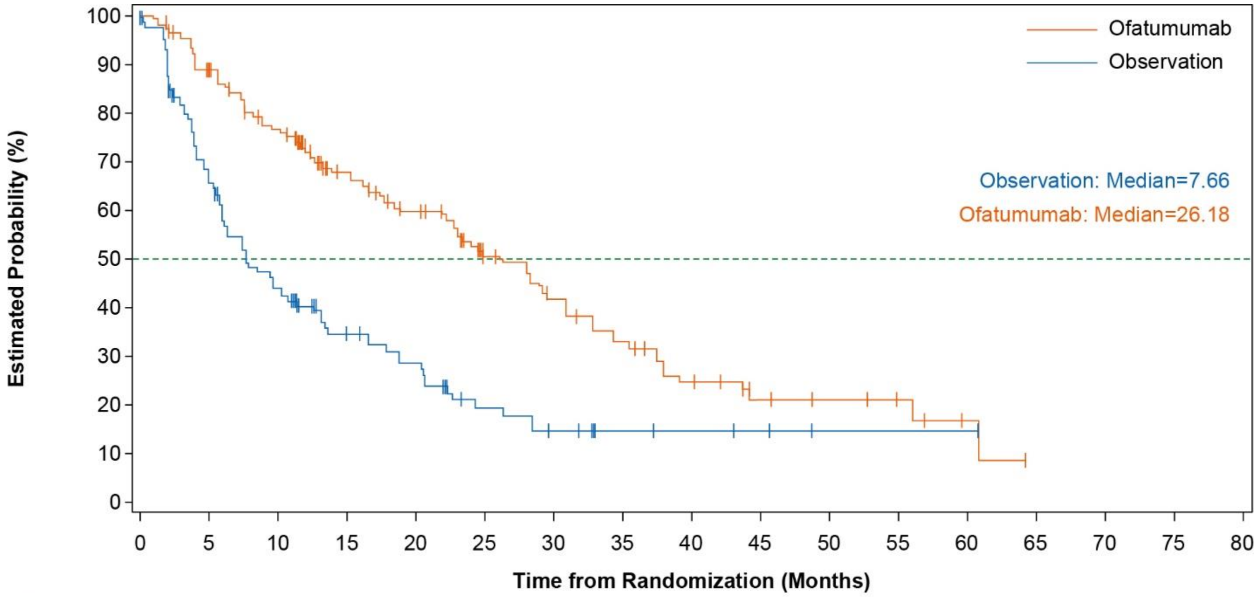

Number of Subjects at Risk:

Ofatumumab	139	117	97	74	63	46	35	26	17	10	7	5	2	0	0	0	0
Observation	107	68	44	29	23	12	8	5	4	3	1	1	1	0	0	0	0

Table S1. Response for lymphocytes (10^9/L) counts for relapsed patients

Summary	Ofatumumab<br>N=239	Observation<br>N=241
N*(%)	100 (41.84)	146 (61.09)
MEAN (Min, Max)	9.26 (0.19, 75.23)	12.82 (0.08, 122.77)
STD	10.07	19.75

*Number of subjects with progression where lab date is +/- 30 days of progression date

STD, standard deviation; Min, minimum; Max, maximum

Figure S2

a)

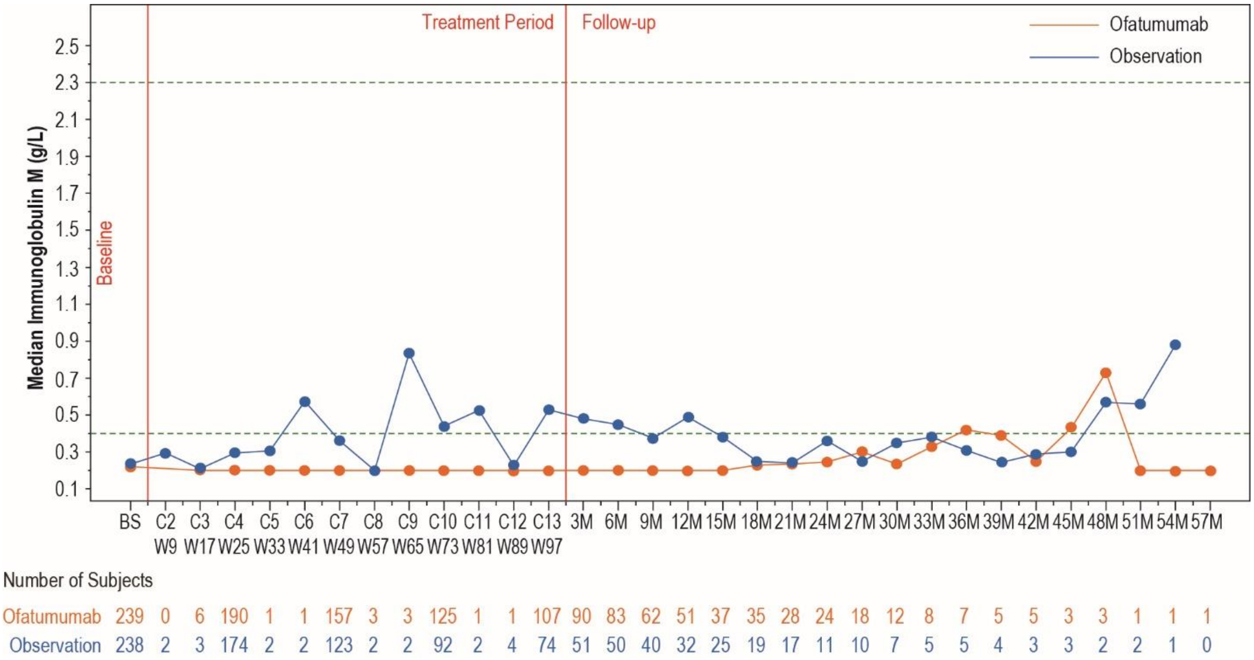

The horizontal lines represents low (0.4 g/L) and high (2.3 g/L) normal ranges

b)

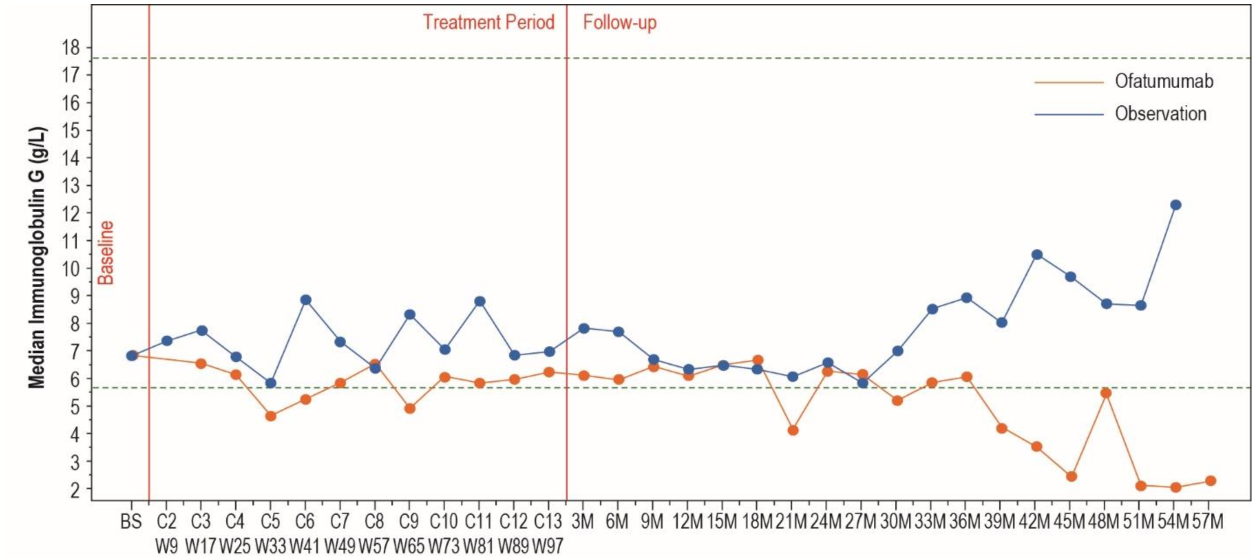

Number of Subjects

	BS	C2 W9	C3 W17	C4 W25	C5 W33	C6 W41	C7 W49	C8 W57	C9 W65	C10 W73	C11 W81	C12 W89	C13 W97	3M	6M	9M	12M	15M	18M	21M	24M	27M	30M	33M	36M	39M	42M	45M	48M	51M	54M	57M
Ofatumumab	239	0	6	190	1	1	157	3	3	125	1	1	107	90	83	62	51	37	35	28	24	18	12	8	7	5	5	3	3	1	1	1
Observation	238	2	3	174	2	2	123	2	2	92	2	4	74	51	50	40	32	25	19	17	11	10	7	5	5	4	3	3	2	2	1	0

The horizontal lines represents low (5.65 g/L) and high (17.65 g/L) normal ranges

c)

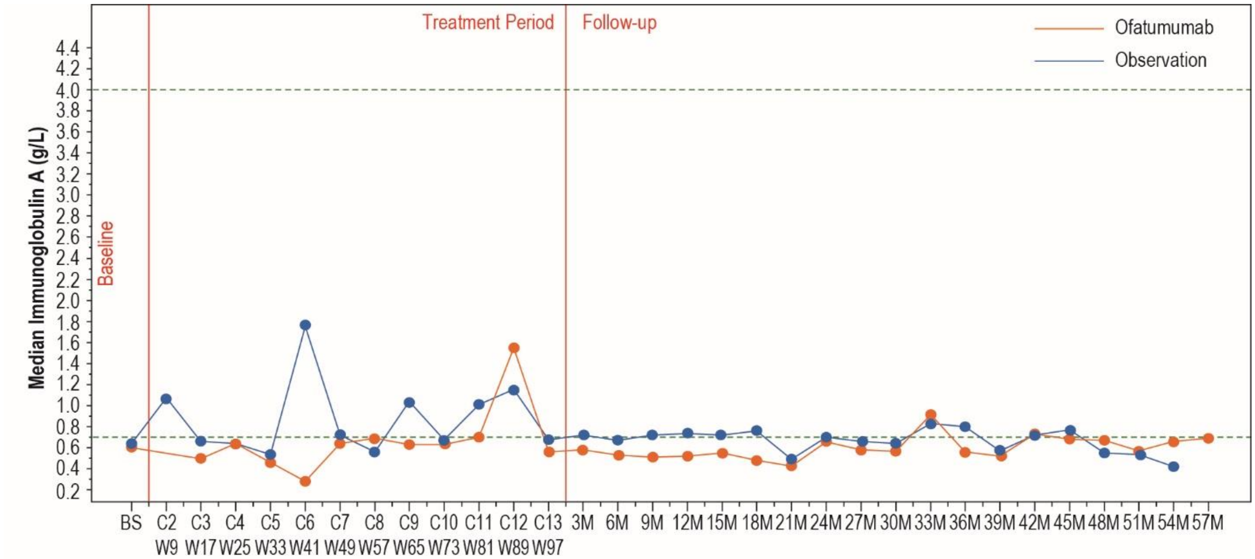

Number of Subjects

	BS	C2 W9	C3 W17	C4 W25	C5 W33	C6 W41	C7 W49	C8 W57	C9 W65	C10 W73	C11 W81	C12 W89	C13 W97	3M	6M	9M	12M	15M	18M	21M	24M	27M	30M	33M	36M	39M	42M	45M	48M	51M	54M	57M
Ofatumumab	239	0	6	189	1	1	157	3	3	125	1	1	107	89	83	62	51	37	35	28	24	18	12	8	7	5	5	3	3	1	1	1
Observation	238	2	3	174	2	2	123	2	2	92	2	4	74	51	50	40	32	25	19	17	11	10	7	5	5	4	3	3	2	2	1	0

The horizontal lines represents low (0.7 g/L) and high (4 g/L) normal ranges
